# Supplementary material for: MetaRibo-Seq measures translation in microbiomes
Source: Nat Commun. 2020 Jun 29;11:3268. doi: 10.1038/s41467-020-17081-z (PMC7324362; doi:10.1038/s41467-020-17081-z)
Supplement: Supplementary file 10 — Supplementary Data 7 [file 41467_2020_17081_MOESM10_ESM.zip › File2/Confidence_High_Taxonomy/85988_out.krona.html]

Javascript must be enabled to view this page.

members
magnitude
magnitudeUnassigned
count
unassigned
taxon
rank

85988\_out

24

superkingdom
24
2

24
phylum
1239

class
24
186801

24
order
186802

family
24
186806

24
genus
1730

24
species
1262889

SRS014613\_contig\_number\_10080SRS015578\_contig\_number\_1632SRS015890\_contig\_number\_14123SRS016335\_contig\_number\_43316SRS019068\_contig\_number\_contig-100\_914.363859SRS042284\_contig\_number\_5947SRS043001\_contig\_number\_contig-100\_13039.13039SRS045645\_contig\_number\_8315SRS047014\_contig\_number\_contig-100\_19694.53438SRS048164\_contig\_number\_contig-100\_24229.24229SRS050925\_contig\_number\_contig-100\_18905.18905SRS052027\_contig\_number\_9548SRS063040\_contig\_number\_contig-100\_1432.103273SRS077194\_contig\_number\_6703SRS078242\_contig\_number\_contig-100\_16403.54024SRS098514\_contig\_number\_contig-100\_1302.1302SRS098644\_contig\_number\_14094SRS1041144\_contig\_number\_6448SRS104485\_contig\_number\_2825SRS140492\_contig\_number\_contig-100\_1167.84610SRS142503\_contig\_number\_39911SRS143070\_contig\_number\_29SRS147346\_contig\_number\_60381SRS148784\_contig\_number\_contig-100\_25087.25088
